# Lipid-Shell PARCH: A Physically Motivated Scale for Transmembrane Residue Hydropathy

**DOI:** 10.1021/acs.jpcb.6c03679

**Published:** 2026-08-13

**Authors:** Ratnakshi Mandal, Shikha Nangia

**Affiliations:** Department of Biomedical and Chemical Engineering, 2029 Syracuse University, Syracuse, New York 13244, United States

## Abstract

Understanding how amino acid residues partition from water into lipid bilayers is fundamental to membrane protein folding, stability, and function. Existing hydrophobicity scales derive from isolated protein systems, organic solvent approximations, or computationally expensive free energy methods, each with significant limitations in capturing the full thermodynamic and topographic complexity of membrane protein environments. Here we present a lipid-shell modification to the protocol for assigning a residue’s character on a hydropathy (PARCH) scale, in which the protein’s first hydration shell is enclosed by a lipid boundary layer during thermal annealing. This modification preserves the core PARCH methodology—evaluating water retention around residues as a function of temperature—while imposing the chemical potential boundary condition appropriate for membrane-embedded proteins. Using the OmpLA host–guest system, we compute PARCH values (PVs) that align with the experimental water-to-bilayer transfer free energy scale of Moon and Fleming (PNAS, 108, 10174–10177, 2011) without calibration to that data. We further demonstrate that depth-dependent PV profiles for arginine and leucine mirror experimentally measured partition energies across six membrane positions. Finally, PVs for a tandem arginine double mutant reveal a per-residue cooperative hydration redistribution that provides microscopic insight into thermodynamic cooperativity previously measured experimentally. Together, these results establish the lipid-shell PARCH modification as a computationally affordable and physically meaningful approach to quantifying membrane hydropathy that is sensitive to residue identity, membrane depth, and cooperative hydration among tandem charged residues.

## Graphical abstract

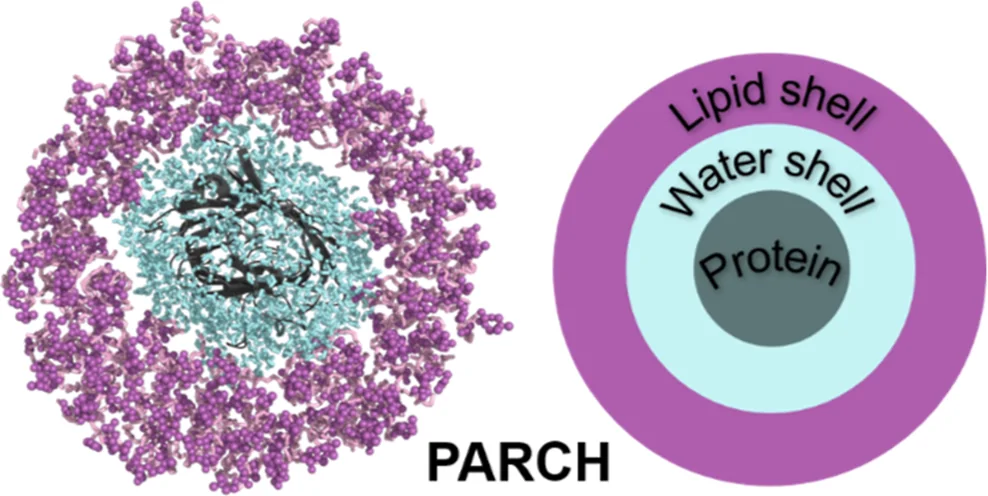

## Introduction

Membrane proteins constitute roughly 30% of all proteins encoded in the human genome^1,2^ and are central to processes including signal transduction, ion transport, and molecular recognition.^3^ Despite their biological importance, the physicochemical principles governing how these proteins fold^4^ and stabilize within lipid bilayers remain incompletely understood. A critical component of membrane protein stability is the energetic cost or benefit of placing each amino acid side chain into the hydrophobic interior of a lipid bilayer^3–6^ —a quantity that depends not only on the chemical identity of the side chain but on the depth at which it resides within the membrane, the local protein topography, and the composition of the surrounding lipid environment.^7^

In the membrane protein literature, the outer membrane phospholipase A (OmpLA) from *Escherichia coli* has emerged as a powerful experimental scaffold for quantifying membrane insertion energetics.^8,9^ OmpLA is particularly valuable because it can spontaneously fold and insert into lipid bilayers from an unfolded, water-solvated state. This property makes it an ideal host–guest system—a well-defined thermodynamic reference frame in which individual amino acid substitutions can be introduced at specific membrane depths and their energetic consequences measured. Unlike many membrane proteins whose insertion depends on translocon machinery, OmpLA undergoes autonomous membrane insertion, allowing measurements in this system to directly report on the water-to-bilayer transfer process that underlies membrane protein folding and stability.^10^

Understanding water-to-bilayer transfer energetics has implications that extend well beyond protein folding. The ability of charged residues to partition into the membrane interior is central to the mechanism of voltage-gated ion channels, where arginine-rich voltage sensor helices must traverse the hydrophobic core during gating.^11–13^ The pH-dependent membrane partitioning of acidic residues is relevant to the action of bacterial toxins^14^ and to the behavior of membrane-active peptides in tumor microenvironments characterized by acidic extracellular pH.^15^ Accurate quantification of these transfer energetics also underpins the development of computational tools for transmembrane helix prediction, force field parametrization, and structure-based drug design targeting membrane proteins.

In 2011, Moon and Fleming^16^ reported an experimental water-to-bilayer transfer free energy scale derived from a native transmembrane protein partitioning into a phospholipid bilayer using OmpLA as a host–guest scaffold. Their measurements revealed that the hydrophobic effect contributes equally to membrane and soluble protein stability, that arginine can be accommodated near the bilayer midplane at substantially lower energetic cost than MD simulations predict, and that tandem arginine residues near the midplane exhibit thermodynamic cooperativity consistent with shared water penetration or membrane deformation. These findings represent the most physically rigorous experimental benchmark currently available for the energetics of water-to-bilayer amino acid transfer.

More recently, we developed the protocol for assigning a residue’s character on a hydropathy (PARCH) scale, a computationally affordable, topography-sensitive method for quantifying amino acid hydropathy in the context of folded proteins.^17^ PARCH evaluates the collective response of water molecules in the protein’s first hydration shell to increasing temperatures during a thermal annealing simulation. Residues that readily shed water during annealing are assigned low PARCH values (hydrophobic), while residues that retain water are assigned high values (hydrophilic). A key strength of PARCH is its sensitivity to nanoscale topography—the local geometric context of bumps, crevices, pockets, and channels that modulate water retention independent of side chain chemistry—a feature absent from fixed residue-based scales. PARCH has been successfully applied to soluble proteins, membrane proteins, self-associating proteins,^17,18^ nucleic acids,^19^ nucleic acid–protein complexes,^19^ nucleosomes,^20^ and proteins with post-translational modifications,^21^ has been extended across multiple water models.^22^

However, a fundamental limitation of the original PARCH framework for membrane protein applications is that the water shell surrounding the protein is bounded by effectively open space during annealing, rather than by the lipid environment that constitutes the actual chemical boundary for transmembrane residues. This means that for lipid-facing residues in the transmembrane domain, water evaporation during annealing encounters no lipid barrier—an unphysical boundary condition that may underestimate the degree to which the lipid environment modulates local hydration.

Here we address this limitation by introducing a lipid-shell modification to the PARCH protocol, in which a shell of lipid molecules surrounds the protein–water system during thermal annealing. This modification does not alter the core PARCH methodology: water molecules in the hydration shell are still the probe of residue hydropathy, and their retention or evaporation during annealing still constitutes the PARCH signal. Rather, the lipid shell imposes the correct chemical boundary condition for membrane-embedded residues, such that water molecules at the protein–lipid interface must overcome a hydrophobic barrier to evaporate, modulating their retention in a physically meaningful way. We apply this lipid-shell PARCH framework to the OmpLA host–guest system of Moon and Fleming,^16^ compute PVs for all 20 amino acids at position 210, examine depth-dependent PV profiles for arginine and leucine across six membrane positions, and analyze cooperative hydration effects in a tandem arginine double mutant. Our results demonstrate strong qualitative agreement with experimental water-to-bilayer transfer free energies, depth-dependent partitioning profiles, and thermodynamic cooperativity, establishing lipid-shell PARCH as a powerful and affordable complement to experimental and computational approaches for membrane hydropathy quantification.

## Methods

### PARCH Methodology

The PARCH scale evaluates amino acid hydropathy by monitoring the thermal response of water molecules in the protein’s first hydration shell during a simulated annealing protocol.

The protein is placed in a shell of water, and the system is subjected to a stepwise temperature ramp. At regular intervals, the number of water molecules retained within a specified cutoff distance from each residue is recorded. These water count data are then used to compute the time-averaged autocorrelation function ${\mathrm{C̅}}_{i}$ for each residue *i*. The corresponding PARCH value (*PV*_*i*_) is subsequently computed as

1 $${PV}_{i}=\frac{{\mathrm{C̅}}_{i}}{{\mathrm{C̅}}_{ref}}\times 10$$

where ${\mathrm{C̅}}_{ref}$ is the time-averaged autocorrelation function of the single zwitterionic lysine residue, which serves as the reference standard. The rationale for using the time-averaged autocorrelation function and the selection of zwitterionic lysine as the reference has been described in our previous work.^17–22^

### Lipid-Shell Modification

In the lipid-shell modification introduced here (Figure 1), the protein–water system is enclosed by a shell of lipid molecules prior to the PARCH annealing protocol. The lipid shell consists of a mixture of 1,2-dioleoyl-*sn*-glycero-3-phosphocholine (DOPC) and 1,2-dipalmitoyl-*sn*-glycero-3-phosphocholine (DPPC). Lipid molecules are position-restrained throughout and are not tracked as part of the PARCH signal—their sole function is to impose a hydrophobic boundary condition at the protein–lipid interface, replacing the unphysical open-space boundary of the original protocol with the chemical potential environment experienced by transmembrane residues in a real bilayer. Water molecules remain the exclusive PARCH probe. All other annealing parameters, such as the temperature range, step size, hydration shell thickness, time step, and PV normalization, are identical to our original PARCH approach described earlier.

**Figure 1:**
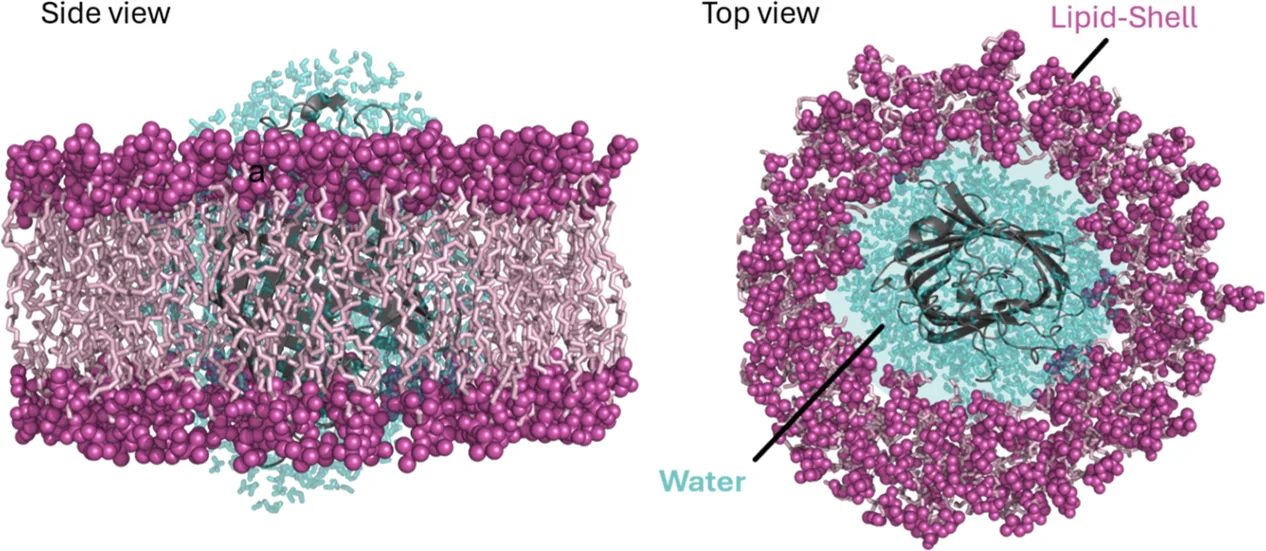
Lipid-shell PARCH system for OmpLA. Side view (left) and top-down view (right) of the simulation system used for lipid-shell PARCH calculations. OmpLA (gray) is enclosed by an annular lipid shell (5 nm radius) consisting of DOPC/DPPC molecules, with phosphocholine headgroups rendered as magenta spheres and acyl chains shown as pink sticks. Water molecules (teal) occupy the OmpLA’s first hydration shell.

### OmpLA System

We employed the outer membrane phospholipase A (OmpLA) as the transmembrane scaffold, following the design of Moon and Fleming.^16^ The alanine at sequence position 210 was substituted with each of the other 19 natural amino acids, generating 19 sequence variants in addition to the wild-type (WT). Position 210 is a lipid-facing exterior residue whose α-carbon is located 0.2 Å from the midplane of OmpLA’s transmembrane region, making it ideal for probing the hydrophobic core environment of the bilayer. The three-dimensional structure of OmpLA (PDB ID: 1QD5) was used as the starting coordinates for all simulations. All mutations were performed using PyMOL. These mutations are illustrated in Figure 2.

**Figure 2:**
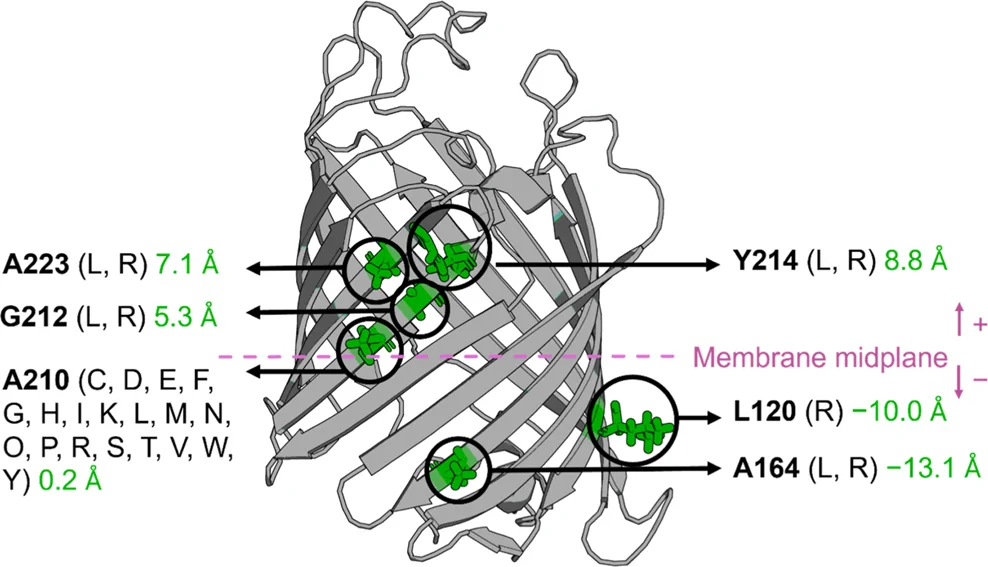
Membrane depth of mutated residues in OmpLA. The OmpLA β-barrel (gray cartoon) is shown in cross-section with the membrane midplane indicated by the dashed magenta line. WT residues at the six substitution sites are shown in green, with their α-carbon distances from the midplane labeled (Å).

### Depth-Dependent Analysis

To examine the effect of membrane depth on residue hydropathy, arginine and leucine mutations were introduced at five additional positions in the OmpLA sequence (positions 120, 164, 212, 214, and 223), and lipid-shell modified PARCH values were compared, following the experimental design of Moon and Fleming.^16^ The membrane depths of the α-carbons at these positions, measured as distance from the bilayer midplane along the membrane normal, are −10.0 Å (position 120), −13.1 Å (position 164), 0.2 Å (position 210), 5.3 Å (position 212), 8.8 Å (position 214), and 7.1 Å (position 223). Positions 164 and 214 are closest to the membrane–water interface, while positions 210 and 212 are nearest the hydrophobic midplane. The membrane depth of each residue of interest was determined by fitting the principal axis of the OmpLA barrel using singular value decomposition (SVD) of all α-carbon coordinates. The centroid of the α-carbon positions was first calculated and used to center the coordinate system. The first principal component of the centered coordinates, determined by SVD, defines the barrel axis—the line running through the geometric center of the transmembrane barrel along the membrane normal. Each residue’s membrane depth was then calculated as the projection of its α-carbon position onto this axis, referenced to position 210, whose α-carbon projects to within 0.2 Å of the barrel midplane, consistent with the crystallographic analysis of Moon and Fleming.^16^ The different amino acids are shown in Figure 2.

### Double Arginine Cooperative Analysis

To examine cooperative hydration effects between tandem charged residues, a double arginine variant (A210R, G212R) was constructed and analyzed. PVs were computed independently for positions 210, 211, and 212 in the WT, A210R single mutant, G212R single mutant, and A210R/G212R double mutant backgrounds. Position 211 in OmpLA’s native sequence is occupied by lysine, providing a naturally occurring charged residue between the two engineered arginine positions.

## Results

### Δ*PV* Recapitulates the Moon–Fleming Hydrophobicity Ordering

We emphasize that PARCH values and the Moon–Fleming ΔΔ*G*_w,l_^°^scale report on fundamentally different observables—water retention during thermal annealing versus equilibrium transfer free energy—and are not related by a direct thermodynamic equation. Both, however, are governed by the same underlying balance of residue-water and residue-lipid interactions, and are therefore expected to correlate monotonically. To test this, we computed Δ*PV* = *PV*(AA) – *PV*(Ala) for all 20 amino acids at position 210 of OmpLA and compared the result directly to the Moon–Fleming ΔΔ*G*_w,l_^°^ scale (Figure 3). Both panels share the same *x*-axis ordering and the same alanine-referenced zero baseline, making tier correspondence immediately apparent by vertical inspection.

**Figure 3:**
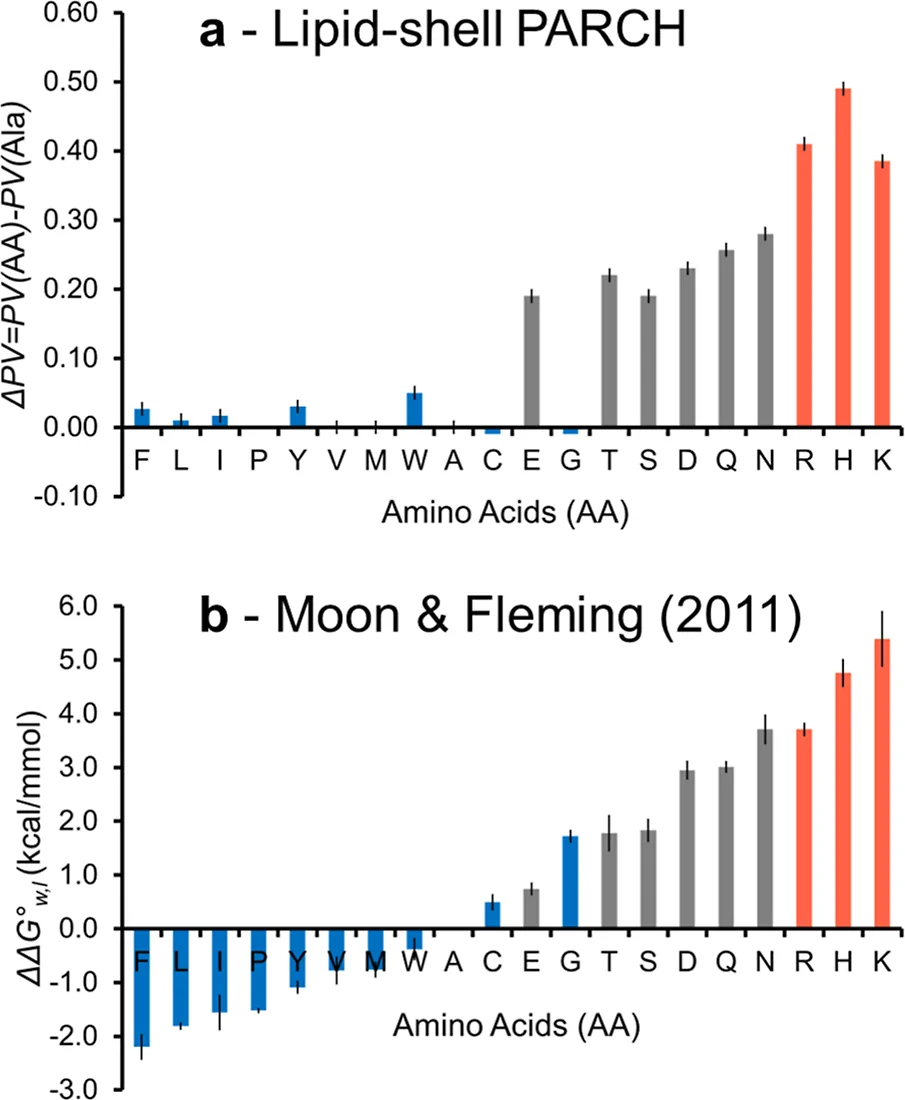
Lipid-shell PARCH recapitulates the water-to-bilayer hydrophobicity hierarchy of Moon and Fleming (2011). (a) Lipid-shell PARCH values expressed as Δ*PV* = *PV*(AA) – *PV*(Ala) for all 20 amino acids substituted at position 210 of OmpLA. (b) Experimental water-to-bilayer transfer free energies (ΔΔ*G*_w,l_°, kcal mol^–1^) from Moon and Fleming (2011), referenced to alanine. Amino acids are ordered identically in both panels following the Moon and Fleming sequence. Color coding reflects the three-tier classification derived from natural gaps in the Δ*PV* distribution: blue—Tier 1 hydrophobic (Δ*PV* ≤ 0.08; ΔΔ*G*_w,l_° ≤ 0); gray—Tier 2 amphiphilic (Δ*PV* 0.10–0.30); orange—Tier 3 hydrophilic (Δ*PV* ≥ 0.35).

### Three-Tier Classification

Examination of the full Δ*PV* distribution reveals two natural gaps that define three physically meaningful tiers without reference to the Moon–Fleming data.^16^ A gap between *W* (Δ*PV* = +0.08) and *E* (Δ*PV* = +0.19) separates residues that have hydropathy similar to alanine from those that are more hydrophilic. A larger gap between *N*/*D*/*Q* (Δ*PV* ≈ +0.23–0.28) and *R* (Δ*PV* = +0.42) separates polar uncharged residues from formally charged residues (Figure 3). Because these boundaries emerge from the PARCH data independently, their strong correspondence with the Moon–Fleming thermodynamic ordering validates the physical fidelity of the lipid-shell modification without requiring any calibration to experimental transfer energies.

Tier 1 (hydrophobic, Δ*PV* ≤ + 0.08) comprises F, L, I, P, Y, V, M, W, A, C, and G—all residues with negative or near-zero ΔΔ*G*_w,l_^°^ in Moon–Fleming.^16^ Negative Δ*PV* values for C and G confirm both are more hydrophobic than alanine at the midplane: glycine presents less surface for water coordination than alanine’s methyl group; cysteine’s thiol is weakly hydrating in the hydrophobic core. Tier 2 (amphiphilic, Δ*PV* +0.10–0.30) comprises E, T, S, D, Q, and N—polar but uncharged residues whose hydrogen-bonding capacity allows meaningful water coordination at the midplane without the electrostatic demand of a full charge. PARCH captures the Moon–Fleming spread (+0.5 to +1.9 kcal mol^–1^), reflecting saturation of water retention capacity for polar uncharged residues at the hydrophobic midplane. Tier 3 (hydrophilic, Δ*PV* ≥ + 0.35) comprises only R, H, and K—the formally charged residues—separated from the rest of the distribution by a gap of more than 0.14 Δ*PV* units.

Three additional differences between the scales are noted: (i) *G* shows Δ*PV* ≈ 0 (Tier 1) but ΔΔ*G*_w,l_^°^ = 1.72 ± 0.12 kcal mol^–1^ likely reflecting backbone conformational effects not captured by side-chain water retention; (ii) aromatic residues F, Y, and W rank Tier 1 thermodynamically but show slightly elevated Δ*PV* due to π-electron water retention, a topographic effect invisible to bulk thermodynamic measurements; (iii) within-tier reordering is expected given that the two methods measure fundamentally different observables, and this does not represent disagreement.

### Depth-Dependent Δ*PV* Profiles Mirror Experimental Partition Energies

#### Leucine: A Uniformly Hydrophobic Internal Control

Leucine PARCH values are near zero across all six membrane depths (Table 1), confirming that the lipid-shell boundary condition does not artificially modulate water retention for strongly hydrophobic residues as a function of depth. The slight elevations at residues 120 (*PV* = 0.07) and 223 (*PV* = 0.03) are within the noise of the measurement and do not alter the conservative interpretation that leucine is uniformly hydrophobic throughout the bilayer, regardless of membrane position. This is consistent with Moon and Fleming’s observation that leucine partition energies are favorable throughout the membrane with modest depth-dependence. The flat leucine profile is a necessary internal control: a PARCH method that showed depth-dependence for hydrophobic residues would be incorrect. The absence of such an artifact validates the fidelity of the lipid-shell boundary condition.

**Table 1:** Depth-Dependent Lipid-Shell PARCH Values for Leucine and Arginine Substitutions in OmpLA^a^

		PV	
residue	depth (Å)	leucine	arginine
214	8.8	0.04 ± 0.00	0.65 ± 0.10
223	7.1	0.03 ± 0.01	0.43 ± 0.03
212	5.3	0.02 ± 0.00	0.42 ± 0.08
210	0.2	0.02 ± 0.00	0.42 ± 0.06
120	–10.0	0.07 ± 0.00	0.96 ± 0.02
164	–13.1	0.00 ± 0.00	1.20 ± 0.01

a PARCH values for leucine and arginine substituted at six positions in the OmpLA β-barrel based on the distance from the membrane midplane. Membrane depth is measured as the α-carbon distance from the midplane along the membrane normal; negative values indicate positions below the midplane.

#### Arginine: Depth-Sensitive Hydration Mirrors the Experimental Partition Energy Curve

In marked contrast, arginine *PV* values show strong and systematic depth-dependence. The highest *PV* values are observed at positions of residues 164 (*PV* = 1.20) and 120 (*PV* = 0.96), where arginine has relatively free access to bulk water and its intrinsic hydrophilicity is expressed fully. The values decrease for residue positions 210, 212, and 223 (*PV* ∼ 0.42), where the lipid shell most suppresses water access. Position 214, at the distance of 8.8 Å from the membrane midplane shows a much higher *PV* of 0.65 ± 0.10.

The depth-dependent profile directly mirrors the arginine partition energy reported by Moon and Fleming, where ΔΔ*G*_w,l_^°^ is most unfavorable at the midplane and decreases toward the interface. The two measurements report complementary observables: Moon and Fleming measure the thermodynamic cost of placing arginine at each depth (most costly at the midplane), while *PV* measures water retention around arginine at each depth (least water retained at the midplane). Both these scales capture the same physical phenomenon—the hydrophobic core simultaneously maximizes the energetic penalty for arginine insertion and minimizes the water available to solvate its charged side chain. Critically, this depth sensitivity emerges from the lipid-shell geometry alone because no depth-dependent parametrization is introduced, demonstrating that the anisotropic chemical environment of the bilayer is encoded in the physical boundary condition rather than fitted to experimental observations.

### Cooperative Hydration in the Double Arginine Variant Reveals Per-Residue Redistribution

To investigate cooperative hydration effects between tandem charged residues near the bilayer midplane, we computed PVs for positions A210, K211, and G212 across four systems: WT OmpLA, the A210R single mutant, the G212R single mutant, and the A210R/G212R double mutant. The autocorrelation curves ( ${\mathrm{C̅}}_{i}$ defined in eq 1) are shown in Figure 4 for 210–212 positions in the four systems, and the resulting PARCH values are summarized in Table 2. The data reveal a structured redistribution of water retention across the 210–212 positions, and taken together, these provide a mechanistic insight into the thermodynamic cooperativity previously reported by Moon and Fleming.^16^

**Figure 4:**
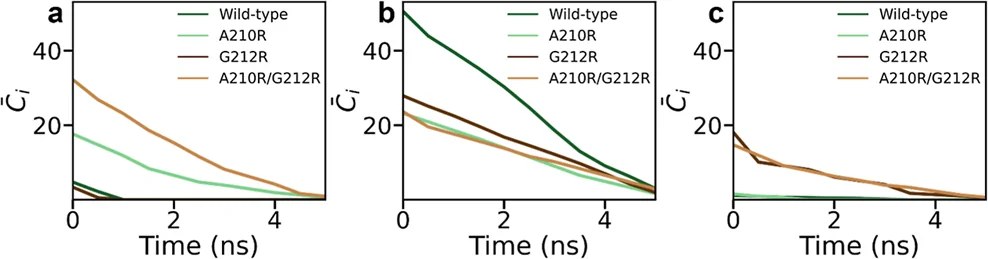
Water autocorrelation functions during the simulated annealing process for residues at (a) position 210, (b) position 211, and (c) position 212 in the OmpLA wild-type (WT) and mutant systems. Dark green: WT; light green: A210R; light brown: A210R/G212R; and dark brown G212R.

**Table 2:** Cooperative Hydration Redistribution in Arginine Variants of OmpLA^a^

		PARCH values			
position	AA (WT)	WT	A210R	G212R	A210R/G212R
210	A	0.01	0.42	0.01	0.73
211	K	1.36	0.79	0.89	0.65
212	G	0.01	0.02	0.42	0.50

a PARCH values of residues for wild-type (WT) protein and three different mutations—A210R, G212R and double mutant A210R/G212R. The PARCH values of the mutated residue are shown in bold.

### WT Baseline

In the WT sequence A210 and G212 exhibit relatively small area under the ${\mathrm{C̅}}_{i}$ (Figure 4a) and PVs of 0.01, consistent with their chemical character and location at the membrane midplane. The native K211, however, displays a strikingly high area under the ${\mathrm{C̅}}_{i}$ curve (Figure 4b) and *PV* of 1.36, indicating that this charged residue maintains substantial water retention even at the midplane. Structurally, position 211 is an inward-facing residue—its backbone remains accessible to the exterior water shell, but its lysine side chain points into the barrel lumen rather than toward the lipid–water interface. The high PV of the native lysine therefore reflects a functionally significant hydration of the barrel interior at the midplane, which may contribute to maintaining the electrostatic environment required for catalytic residue positioning on OmpLA’s outer transmembrane surface and to stabilizing the barrel architecture against the hydrophobic stress of the midplane environment. Position A210, by contrast, is outward-facing—both its backbone and side chain are directly exposed to the exterior water shell and lipid boundary, making it the most lipid-accessible of the three positions examined.

### Single Mutants

Introduction of arginine at position 210 alone (A210R) increases the area under the ${\mathrm{C̅}}_{i}$ curve (Figure 4a) and raises the PV at that position from 0.01 to 0.42, reflecting the strong hydrophilicity of arginine at the midplane and its capacity to recruit water from the lipid–water interface into the hydrophobic core. Notably, as shown in Figure 4, this single substitution also reduces the area under the ${\mathrm{C̅}}_{i}$ curve and reduces the PV of the neighboring lysine at position 211 from 1.36 to 0.79, while position 212 glycine remains essentially unaffected at 0.02 (Table 2). The reduction in lysine PV upon introduction of a single outward-facing arginine at 210 suggests that even one flanking arginine begins to reorganize the local hydration environment, partially redistributing water retention away from the inward-facing lysine and toward the lipid–water interface.

The G212R single mutant produces a symmetric response—position 212 rises from 0.01 to 0.42, matching the A210R result at position 210 precisely, while the lysine at 211 again decreases, this time to 0.89. These trends are also reflected by the area under the ${\mathrm{C̅}}_{i}$ curves (Figure 4b,c). It is notable that the inward-facing G212R achieves the same *PV* as the outward-facing A210R despite its side chain pointing into the barrel interior rather than toward the exterior water shell. This indicates that the inward-facing arginine side chain retains water from the barrel interior environment through the protein scaffold, achieving equivalent hydration to the outward-facing arginine despite drawing on a geometrically distinct water source. Position 210 alanine remains at 0.01, confirming that the perturbation does not propagate across the lysine to the distal position in the single mutant context. The symmetry between the two single mutant responses is internally consistent and validates the robustness of the lipid-shell PARCH measurements.

### Double Mutant and Cooperativity

The A210R/G212R double mutant reveals two simultaneous cooperative effects. First, both arginine positions show substantially elevated *PV*s relative to their single mutant values: position 210 increases from 0.42 to 0.73, and position 212 increases from 0.42 to 0.50. The area under the ${\mathrm{C̅}}_{i}$ curve is the highest at position 210 (Figure 4a). The asymmetry between the two positions is mechanistically informative. Position 210, outward-facing and directly exposed to the lipid–water interface, gains substantially more from cooperativity than the inward-facing position 212. This reflects their geometric distinction—the outward-facing arginine at 210 has unrestricted access to the exterior water shell and lipid boundary, allowing it to draw more extensively on cooperative water stabilization, while the inward-facing arginine at 212 is partially constrained in its capacity to recruit additional water even in the cooperative context.

The most functionally revealing aspect of the double mutant result is the behavior of the native lysine at position 211. Across the full series, its *PV* decreases monotonically: 1.36 (WT) → 0.89 (G212R) → 0.79 (A210R) → 0.65 (A210R/G212R). In the WT, lysine is the dominant water-retaining residue at this midplane site, maintaining barrel interior hydration through its inward-facing side chain. As flanking arginine residues are introduced one by one, and most substantially when both are present, the lysine progressively relinquishes its hydration burden. In the double mutant, the two flanking arginine residues assume the primary water-retaining role—drawing on the lipid–water interface and barrel interior respectively—while the lysine at 211 becomes relatively more membrane-compatible, with a PV of 0.65 substantially lower than its WT value of 1.36.

This progressive transfer of hydration burden away from the central inward-facing lysine and toward the flanking arginine residues provides a per-residue microscopic explanation for the thermodynamic cooperativity reported by Moon and Fleming,^16^ who demonstrated that the energetic cost of inserting the A210R/G212R double mutant into the membrane is 1.6 kcal/mol less than the sum of the two single mutant insertion costs. In Moon and Fleming’s framework, the double mutant protein inserts into the membrane more easily than would be predicted from the individual single mutants—our lipid-shell PARCH results reveal why: the cooperative hydration arrangement between the two flanking arginine residues renders the central lysine at 211 more membrane-compatible by redistributing the local hydration burden, collectively lowering the energetic cost of the entire three-residue stretch residing at the midplane simultaneously.

This interpretation also connects the cooperative hydration result to OmpLA’s enzymatic function. The native hydration architecture—high-PV inward-facing lysine flanked by near-zero aliphatic residues—represents a functionally optimized balance between barrel interior hydration and outer surface hydrophobicity that is likely important for the structural and electrostatic prerequisites of OmpLA’s dimerization-dependent catalytic mechanism. The cooperative reorganization observed upon double arginine substitution is therefore not merely a model system observation but a perturbation of a functionally meaningful native hydration pattern, underscoring the capacity of lipid-shell PARCH to reveal hydration architecture that is invisible to both bulk thermodynamic measurements and traditional hydrophobicity scales.

## Discussion

The lipid-shell modification to PARCH introduced here addresses a physically motivated limitation of the original protocol for membrane protein applications, while preserving the computational affordability and topographic sensitivity that distinguish PARCH from existing hydropathy quantification approaches. The core innovation is conceptually simple—enclosing the protein–water annealing system within a lipid shell imposes the correct chemical potential boundary condition for membrane-embedded residues without altering the water evaporation signal that constitutes the PARCH observable. Yet this simple modification produces results that are quantitatively meaningful, depth-sensitive, and cooperative-effect-aware in ways that neither the original PARCH protocol nor any existing hydrophobicity scale achieves for membrane proteins.

The three-tier classification that emerges from the lipid-shell PARCH Δ*PV* distribution provides a physically interpretable framework for understanding amino acid behavior at the bilayer midplane, and its correspondence with Moon–Fleming thermodynamic ordering validates the method without requiring any calibration to experimental data. Tier 1 (hydrophobic, Δ*PV* ≤ + 0.08) maps cleanly onto residues with favorable or near-zero Moon–Fleming transfer energies; Tier 2 (amphiphilic, Δ*PV* +0.10–0.30) captures polar uncharged residues whose hydrogen-bonding capacity is compressed by the hydrophobic midplane environment; and Tier 3 (hydrophilic, Δ*PV* ≥ + 0.35) isolates the formally charged residues, separated from the rest of the distribution by a gap exceeding 0.14 Δ*PV* units. Because both the tier boundaries and the overall ordering emerge from the PARCH water retention signal independently, this agreement with an orthogonal experimental scale is a genuine cross-validation of the lipid-shell boundary condition rather than a consequence of parameter fitting. The two measurements report fundamentally different observables—lipid-shell PARCH reports water retention during thermal annealing while Moon–Fleming reports thermodynamic transfer free energy from chemical denaturation—and the fact that both resolve the same three physically meaningful groupings confirms that the lipid-shell modification captures the dominant physicochemical determinants of membrane hydropathy. Discrepancies within tiers, such as the elevated Moon–Fleming ΔΔ*G*_w,l_^°^ for glycine relative to its near-zero Δ*PV*, are interpretable as reflections of backbone conformational and topographic effects that the two methods weight differently and constitute additional physical information rather than methodological disagreement.

The depth-dependent PV profiles for arginine and leucine across six membrane positions represent a direct validation of the lipid-shell modification’s sensitivity to the anisotropic chemical environment of the bilayer. Leucine *PV*s are near zero across all depths, consistent with its uniformly aliphatic character and the favorable partition energies reported by Moon and Fleming throughout the membrane. The near-identical leucine *PV*s across all six positions, while seemingly uninformative, are themselves a meaningful result—they confirm that the lipid shell does not artificially inflate *PV*s for hydrophobic residues at any membrane depth, validating the physical fidelity of the boundary condition. Arginine *PV*s show strong and systematic depth-dependence, peaking at interfacial positions where water access is unrestricted and reaching a minimum at the midplane positions where the lipid shell most strongly suppresses water availability. The shape of this profile closely mirrors Moon and Fleming’s experimentally measured arginine partition energy curve,^16^ confirming that the lipid shell boundary condition encodes genuine depth-dependent hydration physics. Importantly, this depth sensitivity emerges naturally from the lipid shell geometry without any explicit depth-dependent parametrization—the method discovers the anisotropy of the bilayer environment from the physical constraint of the lipid boundary alone.

A finding that emerges clearly from the double mutant analysis and that has no precedent in existing hydrophobicity scales is the sensitivity of lipid-shell PARCH to the orientational geometry of residue side chains and its role in shaping cooperative hydration arrangements. Position 210 faces outward toward the lipid–water interface, while positions 211 and 212 face inward with their side chains pointing into the barrel lumen. Despite this geometric distinction, the inward-facing G212R and outward-facing A210R achieve identical *PV*s of 0.42 in their respective single mutants.

This distinction becomes precise in the double mutant. The outward-facing arginine at position 210 shows an increase in *PV* by 0.3 units in the double mutant, while the inward-facing arginine at position 212 gains only 0.08. This asymmetry is not incidental—it directly reflects the geometric constraint on water access for each residue. The outward-facing arginine at 210 has unrestricted access to the lipid–water interface reservoir and can draw extensively on cooperative stabilization, while the inward-facing arginine at 212 is geometrically limited in its capacity to recruit additional water even when a cooperative partner is present. What is significant is not just that both *PV*s increase, but that they increase by different amounts that are physically interpretable from the structural geometry of each position. The cooperativity therefore operates across two geometrically distinct water sources simultaneously—lipid–water interfacial water at position 210 and barrel interior water at position 212—mediated through the protein scaffold. This makes it a genuinely three-dimensional cooperative phenomenon rather than the shared lipid deformation or interfacial water channel that Moon and Fleming theorized from their bulk thermodynamic measurements alone.

The direct consequence of this cooperative arrangement is the progressive suppression of the native lysine *PV* at position 211: 1.36 (WT) → 0.89 (G212R) → 0.79 (A210R) → 0.65 (A210R/G212R). Each arginine introduction partially redistributes hydration away from the central lysine, and both reduce it most substantially. Critically, this lysine *PV* decrease is the per-residue microscopic explanation for the thermodynamic observation of Moon and Fleming that the A210R/G212R double mutant inserts into the membrane 1.6 kcal/mol more easily than the sum of the two single mutants predicts. The cooperative hydration arrangement between the two flanking arginine residues does not merely stabilize the arginine residues themselves—it actively renders the intervening lysine more membrane-compatible by redistributing its hydration burden outward to the flanking positions. The net result is that the entire three-residue stretch at the midplane becomes collectively easier to accommodate in the membrane than individual residue contributions would predict, which is precisely what Moon and Fleming’s thermodynamic cycle measures at the whole-protein level. Lipid-shell PARCH resolves this cooperativity at per-residue resolution, revealing the mechanistic origin of a bulk thermodynamic observation that existing methods could measure but not explain.

The WT PV of 1.36 for the native lysine at position 211 connects this cooperative result to OmpLA’s enzymatic function. This inward-facing charged residue maintains the highest water retention of any position examined across any sequence background, suggesting it plays a structural role in sustaining barrel interior hydration at the midplane that is unlikely to be incidental. OmpLA’s catalytic mechanism requires dimerization-dependent reorganization of the transmembrane surface and calcium-coordinated access of phospholipid substrate acyl chains to a hydrophobic pocket on the outer surface. The native hydration balance—high interior hydration maintained by the inward-facing lysine at 211, outer surface hydrophobicity maintained by flanking aliphatic residues at 210 and 212—may be integral to the structural and electrostatic prerequisites for this mechanism. The fact that lipid-shell PARCH reveals this functionally meaningful hydration architecture from a water retention measurement alone, without prior knowledge of OmpLA’s active site geometry or catalytic requirements, speaks to the method’s capacity to uncover biologically relevant hydration patterns from first principles.

The cooperative hydration redistribution observed here has broader implications for understanding voltage-gated ion channel gating. The S4 helix of KvAP carries multiple arginine residues that must traverse the hydrophobic membrane interior during voltage-dependent gating, and the energetic affordability of this process relative to MD predictions has been a matter of sustained debate. Our results suggest that the per-residue redistribution of hydration burden among clustered charged residues—flanking arginine residues cooperatively stabilizing each other while simultaneously rendering central charged residues more membrane-compatible—may represent a general mechanism by which polyarginine sequences manage the thermodynamic cost of membrane translocation. The geometric asymmetry of the cooperativity further suggests that the specific orientational arrangement of charged residues within S4 helices, where inward and outward facing residues alternate in a helical pattern, may be optimized not just for voltage sensitivity but for maximizing cooperative hydration management during the gating transition.

## Conclusions

We have introduced a lipid-shell modification to the PARCH hydropathy scale that imposes a physically appropriate boundary condition for membrane protein hydropathy calculations by enclosing the protein–water annealing system within a lipid shell. Applied to the OmpLA host–guest system of Moon and Fleming,^16^ the lipid-shell PARCH correctly recovers the broad hydrophobic-to-hydrophilic ordering of all 20 amino acids at the bilayer midplane, reproduces the depth-dependent hydration profile of arginine across six membrane positions, and reveals a per-residue cooperative hydration redistribution in a double arginine variant that provides microscopic insight into the thermodynamic cooperativity previously measured experimentally. These results establish lipid-shell PARCH as a computationally affordable and physically meaningful tool for membrane hydropathy quantification, with sensitivity to the topographic and cooperative effects that distinguish membrane protein environments from those of soluble proteins. Although validated here on the OmpLA host–guest system, the underlying methodology is not specific to OmpLA and is broadly applicable to transmembrane proteins of known structure, positioning lipid-shell PARCH for future application across diverse membrane protein systems.
